# Delayed Facial Paralysis following Uneventful KTP Laser Stapedotomy: Two Case Reports and a Review of the Literature

**DOI:** 10.1155/2014/971362

**Published:** 2014-11-11

**Authors:** P. Révész, Z. Piski, A. Burián, K. Harmat, I. Gerlinger

**Affiliations:** Department of Otorhinolaryngology and Head and Neck Surgery, Medical School, University of Pécs, Munkácsy Mihály Utca 2, Pécs 7621, Hungary

## Abstract

Facial palsy that occurs immediately after middle ear surgery (stapedectomy, stapedotomy, and tympanoplasty) can be a consequence of the local anesthetics and it regresses completely within a few hours. In the case of delayed facial palsy, the alarming symptom occurs several days or even weeks after uneventful surgery. The mechanism of the neural dysfunction is not readily defined. Surgical stress, intraoperative trauma, or laceration of the chorda tympani nerve with a resultant retrograde facial nerve edema can all be provoking etiological factors. A dehiscent bony facial canal or a multiple microporotic fallopian canal (microtrauma or laser effect) can also contribute to the development of this rare phenomenon. The most popular theory related to the explanation of delayed facial palsy at present is the reactivation of dormant viruses. Both the thermal effect of the laser and the elevation of the tympanomeatal flap can reactivate viruses resting inside the ganglion geniculi, facial nerve, or facial nuclei. The authors report the case histories of a 55-year-old female, and a 45-year-old male who presented with a delayed facial palsy following laser stapedotomy. The clinical characteristics, the therapeutic options, and the possibility of prevention are discussed.

## 1. Introduction

Delayed facial palsy occurring some days or weeks following uneventful stapedectomy or stapedotomy is a rare symptom with a favorable prognosis. The phenomenon was first described in 1973 by Althaus and House [1], who observed 5 cases (0.2%) among 2307 stapedectomy cases in their department. Overall, fewer than 40 cases have been reported in the literature. Shea performed 14,449 stapedectomies during four decades of ear surgery and encountered delayed facial paralysis in 11 cases (0.07%) within that period [2]. Ng and Maceri reported the first 2 cases in the literature following laser stapedotomy in 1999 [3].

Facial nerve palsy may occur either immediately or after some delay following stapes surgery. Immediate palsy may be due to local anesthetics and may regress within a few hours. Intraoperative severe surgical trauma or transection of the facial nerve can also cause complete facial paralysis immediately. Bonkowsky et al. [4] observed delayed facial palsy that developed only 48–72 days after surgery, whereas in other literature reports the paralysis began even 5–16 days postoperatively, with complete recovery being achieved within 11–70 days in the vast majority of the cases [3–6].

To date, out of 149 surgeries, 2 cases (0,01%) of delayed facial paralysis have occurred among the KTP laser stapedotomies performed at our department since 2006. Both patients recovered fully. The clinical characteristics, the therapeutic options, and the possibility of prevention are discussed below.

## 2. Case Report 1

A 52-year-old female presented at our department with a bilateral progressive hearing loss in January 2010. Microotoscopic examination showed a normal eardrum and tympanic cavity, and audiometry revealed a conductive hearing loss on both sides; on the right, the average air-bone gap (ABG) was a mean of 34 dB on 0.5-1-2-3 kHz, while on the left side the mean ABG was 32 dB. The Carhart notch was present on both sides, and the mean bone conduction threshold was 5 dB on 0.5-1-2-3 kHz. The stapedial reflex was absent on both sides, while the multiple-frequency tympanogram indicated a resonance frequency of 1000 Hz on the left and of 1100 Hz resonance on the right side.

**Figure 1:**
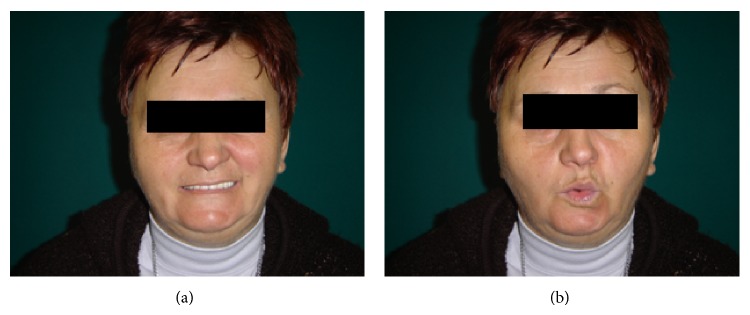
Eight days following stapedotomy, House-Brackmann grade II facial palsy is observed on the right side: teeth showing (a) and lip rounding (b).

Explorative tympanotomy performed because of the suspicion of stapes fixation revealed that the stapes were indeed fixed. KTP laser stapedotomy was carried out on the left side as described earlier [7], whereby the stapes superstructure was removed, and a self-crimping heat memory Nitinol piston was implanted to reconstruct the ossicular chain. The postoperative period was uneventful and the patient was discharged on day 3 following surgery. On postoperative day 8, the patient developed parageusia and pain around the right ear after awakening. House-Brackmann grade II facial palsy later developed on the operated side (Figure 1). No signs of either acute external otitis or acute otitis media were detected, and the blood tests demonstrated normal parameters. Following admission, the patient received 60 mg of methylprednisolone daily (Medrol, Pfizer), which was gradually tapered off in 3 weeks. Acyclovir (Zovirax, Sanofi Aventis) was administered orally for 2 weeks.

**Figure 2:**
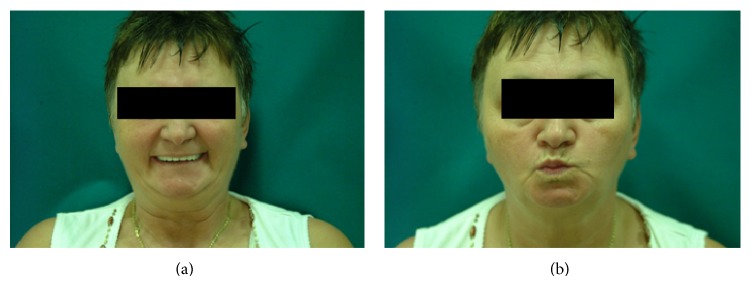
Thirty-six days following stapes surgery: the facial nerve function is intact, teeth showing (a) and lip rounding (b).

The patient gradually recovered, the facial nerve function improved, and the ear pain and the parageusia had regressed by postoperative day 36 (Figure 2). The audiometric evaluation revealed an ABG < 10 dB on 0.5-1-2-3 kHz, while the mean bone conduction threshold had improved by 5 dB relative to the preoperative results.

## 3. Case Report 2

A 45-year-old male developed a progressive bilateral hearing loss in January 2011. His ability to understand speech understanding gradually deteriorated. Examinations in April 2011 suggested stapes fixation, but he refused explorative tympanotomy. At the following visit during the summer of 2013, the microotoscopic examination indicated a normal eardrum on both sides. Audiometry revealed a mixed hearing loss bilaterally; on the right side, the bone conduction threshold was a mean of 20 dB, and the air conduction threshold was a mean of 55 dB (35 dB ABG) on 0.5-1-2-3 kHz, while on the left side the bone conduction threshold was a mean of 25 dB and the air conduction threshold was a mean of 45 dB (20 dB ABG). The Carhart notch was present on both sides (2 kHz). The stapedial reflex was absent bilaterally, while the multiple-frequency tympanogram revealed a resonance frequency of 1250 Hz on the left and of 1750 Hz on the right side.

**Figure 3:**
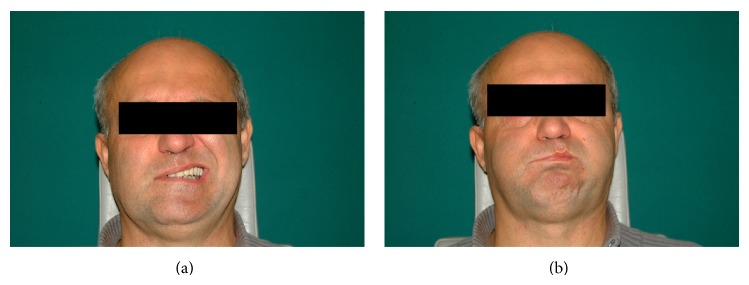
Thirteen days following stapedotomy, House-Brackmann grade III facial palsy is observed on the right side: teeth showing (a) and lip rounding (b).

**Figure 4:**
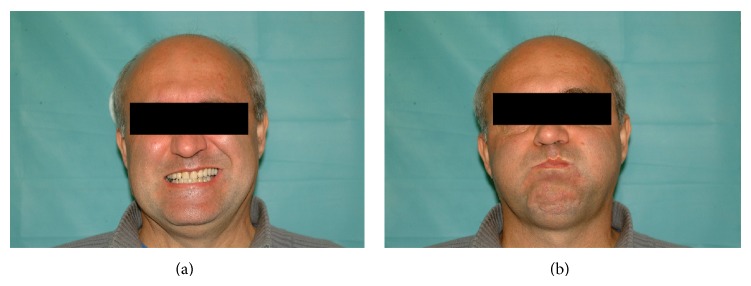
Forty-two days following stapes surgery, the facial nerve function is intact: teeth showing (a) and lip rounding (b).

In September 2013, the patient underwent an uneventful KTP laser stapedotomy on the right side, while the ossicular chain was reconstructed with a self-crimping heat memory NiTiBOND piston. On postoperative day 13, he suffered House-Brackmann grade III facial palsy on the right side while shaving (Figure 3). The symptom was accompanied by a slight pain in the right ear. No signs of either acute external otitis or acute otitis media were detected, and the blood tests showed normal parameters. By postoperative day 42, the patient had recovered completely (Figure 4) following Medrol and Acyclovir therapy administered in the same manner as described in case 1, and the audiometric evaluation revealed a mean of 5 dB ABG on 0.5-1-2-3 kHz, while the mean bone conduction threshold had improved by 5 dB.

## 4. Discussion

Delayed facial palsy occurring a few days or weeks following uneventful stapes surgery is an alarming symptom but generally with a favorable prognosis. There is some disagreement among authors as to the explanation of this phenomenon.

Zohar and Laurian hypothesized that such a Bell palsy-like condition may be provoked by surgical stress, assuming that this was reinforced by the fact that patients presenting with facial palsy generally exhibited trigeminal sensory deficits on the same side [8].

Surgical trauma to a dehiscent facial canal in the tympanic cavity may also lead to edema [1] and consequential paralysis of the facial nerve. Paulsen presented a case where reexploration was indicated due to facial nerve palsy that developed within 24 hours postoperatively; extensive edema in a dehiscent facial canal was reported to have dislocated the implanted prosthesis [9]. Kaplan observed that microscopic dehiscence may occur in the medial part of the facial canal near the oval window, while the bony coverage of the canal is apparently intact. During surgery, tearing of the chorda tympani may lead to retrograde edema, and the heat effect of the laser can additionally provoke edema formation according to the macroscopic dehiscences in cases of laser-assisted stapes surgery [10]. In the past decade however the experience of Gerlinger et al. was somewhat different: they often found that the facial nerve was dehiscent, or laser vaporization of a cholesteatoma sac was requisite near the oval window [7, 11].

In the event of a dehiscent nerve canal, immediate facial palsy is indicative of neurotmesis or axonotmesis following either laser-assisted operations or cold technical manipulations. The favorable prognosis of delayed palsies has demonstrated the presence of neurapraxia [3].

Recent publications suggest that the most reasonable explanation for delayed palsy is the reactivation of dormant viral infections (varicella zoster, herpes simplex 1-2, or Epstein-Barr). Herpes zoster or herpes simplex virus can undergo reactivation for either local or systemic reasons [12]. This explanation seems to be confirmed by the occasional occurrence of facial nerve palsy following acoustic neuroma operations [13]. Surgical stress may provoke herpes simplex reactivation in dermatomes remote from the surgical site [14]. Spine surgeons have reported the reactivation of herpes virus in the surrounding dermatomes following surgery [15]. Viral reactivation may also follow local trauma of the skin branches of the facial nerve subsequent to the elevation of the tympanomeatal flap in any type of ear surgery (tympanoplasty, meatoplasty, etc.). Shea Jr. and Ge reported elevated virus-specific anti-IgG and IgM levels in a patient with delayed facial nerve palsy following stapedectomy, which appears to support the virus theory [16]. Sugita et al. recentlyidentified herpes simplex virus particles in the geniculate ganglion, the facial nerve, and the nuclei of the facial nerve in a novel mouse model [17]. Facial nerve palsy was caused by the inoculation of these viruses through either the posterior part of the auricle or the anterior third of the tongue of the mice via the sensory branches of the facial nerve [17].

Gadolinium-contrast MRI demonstrated significantly higher enhancement in the distal part of the internal ear canal, the labyrinth, the tympanic part and the geniculate ganglion in patients with Bell's palsy, as compared with the control group [18, 19]. Cohen et al. reported corresponding contrast-enhanced MRI results in 2 patients with delayed facial nerve palsy following stapes surgery [5].

Our cases occurred after laser-assisted stapedotomy. Fewer than 40 reports can be found that involve conventional or laser-assisted stapes surgery. The facial nerve canal in patients was apparently intact, and the surgical technique was standard. The laser equipment settings were as usual [11]. The history of the patients did not reveal frequent herpes infections. The lesson learned from our cases is that, besides an appropriate knowledge of laser physics, it is indispensable to apply cooling intraoperatively and to prefer cold instrumentation where a laser is not necessary (e.g., cutting the stapedial tendon). It appears essential to take into account the effect of scattering from the bare bony surface during laser vaporization and coagulation which may result in undesired heating of the facial nerve canal.

Despite the generally favorable prognosis of such cases, we do not rule out the administration of steroid and antiviral medication in the light of the theory of virus reactivation as the explanation of delayed facial nerve palsy. The need for surgical decompression of the facial nerve almost never arises. In cases of complete facial nerve palsy, repeated electroneurography should be considered. Contrast-enhanced MRI is recommended.

The question remains open as to whether or not to administer antiviral treatment preoperatively to a patient with a history of frequent herpes infections [3, 10, 12].

## 5. Conclusions

1. Delayed facial palsy may occur several days or weeks following uneventful stapedectomy, stapedotomy, or tympanoplasty. The prognosis is favorable in almost all cases.
2. Present knowledge suggests that the most reasonable etiology is viral reactivation (herpes zoster, herpes simplex, or Epstein-Barr).
3. In the event of a delayed palsy, acute infection must be ruled out.
4. Gadolinium-contrast MRI is recommended (to confirm tumor formation and higher enhancement in the fundus of the internal ear canal, the labyrinth, and the tympanic part).
5. The lowest possible energy density should be applied, the use of high output should be avoided, and the heat energy generated during laser stapedotomy should be as low as possible.
6. A defocused laser beam may scatter from the bare bony surface and may reach the occasionally osteoporotic facial canal.
7. Gelfoam saturated with cold saline may be beneficial if applied to the facial canal, the round, and the oval window (heat absorption and minimal scattering).
8. The administration of antiviral medication and steroid may be advantageous following the development of delayed facial palsy.
9. Prophylactic antiviral treatment may be considered preoperatively if the patient presents with a history of frequent herpes simplex infections.
